# What about dreams? State of the art and open questions

**DOI:** 10.1111/jsr.13609

**Published:** 2022-04-13

**Authors:** Serena Scarpelli, Valentina Alfonsi, Maurizio Gorgoni, Luigi De Gennaro

**Affiliations:** ^1^ Department of Psychology Sapienza University of Rome Rome Italy; ^2^ Body and Action Lab IRCCS Fondazione Santa Lucia Rome Italy

**Keywords:** activation hypothesis, continuity hypothesis, dream enacting behaviour, dream recall, interindividual differences, nightmares

## Abstract

Several studies have tried to identify the neurobiological bases of dream experiences, nevertheless some questions are still at the centre of the debate. Here, we summarise the main open issues concerning the neuroscientific study of dreaming. After overcoming the rapid eye movement (REM) ‐ non‐REM (NREM) sleep dichotomy, investigations have focussed on the specific functional or structural brain features predicting dream experience. On the one hand, some results underlined that specific trait‐like factors are associated with higher dream recall frequency. On the other hand, the electrophysiological milieu preceding dream report upon awakening is a crucial state‐like factor influencing the subsequent recall. Furthermore, dreaming is strictly related to waking experiences. Based on the continuity hypothesis, some findings reveal that dreaming could be modulated through visual, olfactory, or somatosensory stimulations. Also, it should be considered that the indirect access to dreaming remains an intrinsic limitation. Recent findings have revealed a greater concordance between parasomnia‐like events and dream contents. This means that parasomnia episodes might be an expression of the ongoing mental sleep activity and could represent a viable direct access to dream experience. Finally, we provide a picture on nightmares and emphasise the possible role of oneiric activity in psychotherapy. Overall, further efforts in dream science are needed (a) to develop a uniform protocol to study dream experience, (b) to introduce and integrate advanced techniques to better understand whether dreaming can be manipulated, (c) to clarify the relationship between parasomnia events and dreaming, and (d) to determine the clinical valence of dreams.

## 1 INTRODUCTION

Dreams have been extensively studied from many points of view, focussing on different aspects of the phenomenon. Dreaming is a composite experience occurring during sleep that includes images, sensations, thoughts, emotions, apparent speech, and motor activity. The oneiric production is a form of mental sleep activity that appears strictly related to memory processes and cognitive elaboration (Wamsley & Stickgold, 2010; Mangiaruga et al., 2018). In this respect, some investigations have highlighted that dream features mirror the development of cognitive processes (Mangiaruga et al., 2018; Scarpell et al., 2019a).

Additionally, a growing number of studies have suggested that dream experience might be considered an expression of human wellbeing (Fränkl et al., 2021; Scarpelli et al., 2022) and has a pivotal role in emotional regulation, as suggested by some neurobiological findings (Nielsen & Lara‐Carrasco, 2007). For instance, dream recall and nightmare frequency increase when subjects are exposed to adverse and traumatic events (e.g., Hartmann & Brezler, 2008; Nielsen et al., 2006; Sandman et al., 2013; Tempesta et al., 2013). Also, the qualitative characteristics of dream reports change in parallel with the emotional charge of waking experiences (Schredl, 2006; Scarpelli et al., 2021).

It should be highlighted that psychoanalysis had primacy in dream research until the discovery of the rapid eye movement (REM) sleep stage (Aserinsky & Kleitman, 1953). The interpretation of oneiric contents was one of the main focusses of the Freudian theories positing that dreaming allows access to the unconscious functions of the mind in neurosis treatment (Freud, 1953). Aserinsky and Kleitman (1953) observed specific intervals with rapid and recurrent eye movement and bursts of alpha activity comparable to those that occur during wakefulness. The enthusiasm linked to the discovery of REM sleep considerably influenced dreaming research in several ways, and the neuroscientific study of dreaming is relatively recent. Several studies have attempted to identify the neurobiological bases of dream experience through a neuropsychological approach (Solms, 1997, 2000), neuroimaging (Maquet et al., 1996) and electrophysiological techniques (Marzano et al., 2011; Siclari et al., 2017).

Although several studies provide compelling evidence for the existence of specific brain mechanisms predicting dream recall (e.g., Siclari et al., 2017), many questions are still at the centre of the debate.

The present paper summarises the main open issues concerning the neuroscientific study of dream experience. Specifically, the review offers an overview about (a) the question related to the REM‐non‐REM (NREM) sleep dichotomy, (b) the state–trait‐like problem, (c) the relationship between waking and dreaming state and the manipulation of dreaming, (d) the issue concerning the access to dream experience, (e) the role of nightmares, and (f) the debate on dreamwork in psychotherapy.

### 1.1 The REM‐NREM sleep dichotomy

A classical view of the neurobiological basis of the oneiric activity postulates the existence of a close relationship between dream experience and REM sleep (Hobson et al., 2000; Nielsen, 2000). This hypothesis was based on early electroencephalographic (EEG) observations showing that >70% of individuals awakened during REM sleep reported dreams, while dream recall at the awakening from other sleep stages was rare (Aserinsky & Kleitman, 1955). According to this view, the wake‐like high‐frequency EEG pattern characterising REM sleep would represent the ideal electrophysiological *scenario* for the occurrence of dream experiences, while the slow‐frequency activity characterising NREM sleep would be associated with the absence of oneiric activity. However, using different criteria to collect dream reports, several studies found that successful recall of a conscious experience can be frequently observed also after NREM awakenings, and in a minority of cases no dream experience was reported after REM awakenings (Foulkes, 1962; Nielsen, 2000). Moreover, dream recall is still possible after lesions in brain regions involved in REM sleep generation, while the total disappearance of dream recall can be observed after focal forebrain lesions without an impact on REM sleep (Solms, 2000). Also, dream experience is preserved after pharmacological suppression of REM sleep (Landolt et al., 2001; Oudiette et al., 2012). Finally, dream recall has been recently associated with a similar electrophysiological response after REM and NREM sleep (D'Atri et al., 2019; Siclari et al., 2017). These results suggest that (a) dream and REM sleep are controlled by distinct brain mechanisms, (b) the postulate of a clear distinction between presence and absence of dreaming respectively in REM and NREM has not a solid support, and therefore (c) dreams can occur in any sleep stage.

A dichotomy between NREM and REM sleep has been also hypothesised for the qualitative aspects of dreams. Indeed, it has been proposed that REM and NREM sleep exhibit different kinds of mental activity. According to this view, REM sleep is characterised by an emotional, vivid, and bizarre “dream‐like” mentation (Antrobus, 1983; Casagrande et al., 1996; Foulkes, 1967; Foulkes & Schmidt, 1983; Waterman et al., 1993), while NREM mental activity would be “thought‐like”, with reduced emotional load, greater fragmentation, and contents more similar to waking thoughts (Foulkes, 1967; Rechtschaffen et al., 1963). Nevertheless, the existence of a clear‐cut REM‐NREM dichotomy has been questioned also in this case based on several findings: (a) “dream‐like” reports have been observed also after NREM sleep (Monroe et al., 1965; Solms, 2000; Zimmerman, 1970) and (b) the qualitative differences between REM and NREM dream reports disappear when their length is equated (Antrobus, 1983; Cavallero et al., 1992; Foulkes & Schmidt, 1983).

In light of these observations, the assumption that the presence/absence and the phenomenological aspects of dream experiences strictly depend on the sleep stage per se is simplistic. It is worth noting that a precise definition of the time‐coupling between the sleep stages and the actual occurrence of dream experience is difficult, as the access to sleep mentation is possible only in an indirect way through dream reports after the awakening (see the paragraph “What about direct access to dream experience?”). At the same time, the occurrence of dream experiences in both REM and NREM sleep, two physiological stages characterised by distinct electrophysiological and neurotransmitters patterns, appears paradoxical. Such considerations raised the question of what mechanisms facilitate/inhibit the recall of a conscious experience at the awakening from different sleep stages, and what factors can explain intra‐ and inter‐individual variability in the phenomenology of the oneiric activity.

### 1.2 State‐ and trait‐like facets of dreams

Stable individual characteristics (trait‐like factors) can impact dreams, explaining inter‐individual variability. Sociodemographic factors like gender (Schredl & Reinhard, 2008; Settineri et al., 2019) and age (Mangiaruga et al., 2018; Scarpelli et al., 2019a) can predict dream recall. Interest in dreams (Bealulieu‐Prevost & Zadra, 2007), visual imagery abilities (Cory & Ormiston, 1975), personality dimensions like openness to experience, absorption, psychological boundaries (Beaulieu‐Prevost & Zadra, 2007), and predisposition to suppress negative emotions and thoughts (Malinowski, 2015) appear related to individual differences in the oneiric activity.

Crucially, neuroimaging studies provided evidence about the relationship between dream features and stable brain anatomical and functional characteristics. Qualitative facets of dreams have been associated with volumetric and structural measures of the amygdala‐hippocampus complex in healthy subjects (De Gennaro et al., 2011) and amygdala volume, dorsomedial prefrontal cortical thickness, and dopaminergic activity in patients with Parkinson's disease (De Gennaro et al., 2016). Moreover, compared to low dream recallers, high dream recallers showed (a) greater medial prefrontal cortex white‐matter density (Vallat et al., 2018); (b) higher regional cerebral blood flow in the temporo‐parietal junction during wakefulness, Stage 3, and REM sleep and in medial prefrontal cortex during wakefulness and REM sleep (Eichenlaub et al., 2014a); (c) enhanced functional connectivity within the default mode network (DMN) and between areas of the DMN and memory‐related regions immediately after the awakening (Vallat et al., 2020); and (d) larger event‐related potentials to distracting sounds even during active listening, arguing for enhanced bottom‐up processing of irrelevant sounds but also an enhanced recruitment of top‐down attention as suggested by larger contingent negative variation during target expectancy and P3b to target sounds (Ruby et al., 2021). Taken together, these findings highlight that stable individual features of the brain structure and activation patterns can explain inter‐individual differences in dream experience.

Beyond the influence of trait‐like factors, a growing number of studies also point to the role of the physiological milieu associated with the oneiric experience (state‐like factors). In other words, the specific regional features of the physiological background contingent with dreaming would facilitate or prevent dream recall, potentially explaining intra‐individual differences in dream reports. This possibility has been investigated mainly by assessing the sleep EEG pattern preceding dream recall. In this way, several studies found that a successful dream recall was associated with greater frontal theta oscillations before the awakening from REM sleep (Marzano et al., 2011; Scarpelli et al., 2015; Scarpelli et al., 2019b) and reduced parieto‐occipital alpha activity before the awakening from NREM sleep (Esposito et al., 2004; Marzano et al., 2011). As theta and alpha oscillations are associated with memory processes during wakefulness (Hsieh & Ranganath, 2014), these results suggest that wakefulness and sleep share the same neurobiological mechanisms for the elaboration of episodic memories (see the next paragraph).

On the other hand, a growing number of within‐subject investigations (which allows overcoming the possible influence of stable trait‐like factors) show that a more desynchronised EEG pattern is associated with dream recall in both NREM and REM sleep (Siclari et al., 2017; D'Atri et al., 2019; Scarpelli et al., 2017; Scarpelli et al., 2020a; but see Wong et al., 2020). In particular, dream experience would be facilitated by a pattern of reduced slow‐wave activity (SWA), most steadily in posterior regions (Siclari et al., 2017, 2018). Interestingly, lucid dreams, phenomenon characterised by conscious awareness during the oneiric experience, appear associated with greater EEG gamma activity (Baird et al., 2022; Voss et al., 2009). Furthermore, a transcranial current stimulation delivered in a lower gamma range during REM sleep can affect the ongoing electrophysiological activity and increase self‐reflective awareness in dreams (Voss et al., 2014). These observations are consistent with “activation” theoretical models (Antrobus, 1991; Hobson & McCarley, 1977; Koulack & Goodenough, 1976), which postulate that dream recall would be facilitated by a greater level of arousal during sleep, represented at an electrophysiological level by higher brain activation. Indeed, the frequency of dream recall increases in association with a sleep pattern characterised by greater sleep fragmentation (van Wyk et al., 2019), faster spindles, especially in central and posterior cortical areas (Siclari et al., 2018), intra‐sleep wakefulness (De Gennaro et al., 2010; Eichenlaub et al., 2014b; Vallat et al., 2017), and sleep arousal (Polini et al., 2017; Schredl, 2009). Furthermore, a night of recovery sleep after a period of prolonged wakefulness, usually characterised by reduced awakenings, almost totally abolished dream recall after the final morning awakening (De Gennaro et al., 2010). The SWA represents a marker of sleep intensity (Borbély & Achermann, 1999), likely subserving the fading of consciousness during sleep. Thus, the pattern of local SWA reduction in association with dreaming activity may represent the electrophysiological marker of the greater arousal level needed for a successful dream recall. Moreover, this evidence provides a reliable explanation for the apparently paradoxical occurrence of dreams in states of consciousness (i.e., REM and NREM sleep) characterised by drastically different EEG patterns.

Overall, these findings highlight the crucial role of the physiological state preceding dream recall. However, several questions remain open. First, the influence of circadian and homeostatic factors on the oneiric experience and its electrophysiological pattern is not clear (Chellappa et al., 2011; D'Atri et al., 2019; Scarpelli et al., 2017; Scarpelli et al., 2020a). Moreover, the impact of the regional distribution of SWA on qualitative dream facets needs to be fully investigated, as empirical preliminary evidence has been provided only by Siclari et al. (2017). Finally, the possible interaction between state‐ and trait‐like factors should be carefully considered.

### 1.3 Continuity between waking and dream experience

The above‐mentioned “activation hypothesis” represents one of the main theoretical frameworks on dreaming, along with the so‐called “continuity hypothesis” (Domhoff, 2017; Schredl & Hofmann, 2003). In the early 1970s, Bell and Hall (1971) firstly proposed that waking experiences may have continuity in sleep. The formulation of the original concept has gone through several re‐interpretations and adjustments since then.

Early cognitively‐oriented studies focussed on the continuity between dream contents and waking events, personal concerns, thoughts, behaviours, and emotions, suggesting that waking‐life experiences are reflected into subsequent dreams (Nielsen & Powell, 1992; Schredl, 2006; Blagrove, 2011; Vallat et al., 2017). Compelling evidence also showed the key role of the personal and emotional salience in mediating the preferential incorporation of waking‐life aspects during mental sleep activity (Malinowski & Horton, 2014).

Further, different time intervals between waking experiences and related dream contents could represent “day‐residue effect” or “dream‐lag effect” as a function of the elapsed period (i.e., 1–2 days and 5–7 days, respectively) (Eichenlaub et al., 2017). Specifically, the delayed incorporation of waking life events (“dream‐lag effect”) was selectively observed during REM sleep and for personally significant events (Van Rijn et al., 2015).

A complementary field of study posits the continuity between waking state and mental sleep activity from a neurophysiological perspective. Namely, a growing body of evidence suggests that brain mechanisms underlying cognitive and emotional functioning remain the same across different states of consciousness (e.g., Marzano et al., 2011; Eichenalub et al., 2018).

The involvement of alpha (8–12 Hz) and theta (5–7 Hz) oscillations in memory‐related neural processes during wakefulness are well‐established, especially as regards episodic‐declarative memory (Klimesch, 1999). In particular, the increase in the frontal theta activity and the alpha power decrease during the encoding phase of episodic memories were found to play a pivotal role in the subsequent recall of stored information (Hsieh & Ranganath, 2014; Klimesch, 1999).

Over the last two decades, several studies were conducted under the assumption that dream encoding and recall could represent a peculiar form of episodic memory (Fosse et al., 2003). As previously mentioned, a successful dream recall has been linked to higher frontal theta activity during REM sleep (Marzano et al., 2011; Scarpelli et al., 2015) and lower alpha activity over the temporo‐parietal region during NREM (Esposito et al., 2004; Marzano et al., 2011; Takeuchi et al., 2003). Moreover, the topographical distribution of the above‐mentioned frequency bands resembles brain regions involved in encoding and retrieval mechanisms during wakefulness.

A large body of experimental studies have also shown the continuity between dreaming and emotional processing (for a review, see Scarpelli et al., 2019c). First of all, as described in the previous paragraph, neuroimaging studies showed the relationship between qualitative and quantitative stable aspects of dream experience and structural parameters of limbic areas (De Gennaro et al., 2011). Consistently, subjects reporting higher levels of fear in their dreams showed a concomitant higher activation of the medial prefrontal cortex, responsible for reduced activation of the amygdala, insula, and midcingulate cortex both during sleep and wakefulness (Phelps et al., 2004; Sterpenich et al., 2020). Further, the main brain circuits involved in emotional processing during wake are highly activated during REM sleep, such as the limbic system (Nir & Tononi, 2010) and reward system (Perogamvros & Schwartz, 2012). Notably, a recent simultaneous EEG‐functional magnetic resonance imaging study demonstrated the privileged re‐emergence during sleep of patterns of brain activity associated with a recent rewarding (compared to a non‐rewarding) waking experience during sleep (Sterpenich et al., 2021).

Starting from these findings, many researchers stated that dream activity might have a crucial role in processing emotional events experienced during wakefulness (see Scarpelli et al., 2019c). More in‐depth, the theta (Nishida et al., 2009; Boyce et al., 2016; Sopp et al., 2018) and gamma activities (Van Der Helm et al., 2011) were identified as the EEG markers of emotional memory processing. Selective sleep deprivation protocols provided experimental evidence about the lack of emotional memories consolidation in the absence of REM sleep stage (Spoormaker et al., 2014; Wagner et al., 2001), supporting the notion that dreaming represents the privileged scenario for the offline reprocessing of waking emotional stimuli.

Keeping in mind the unitary perspective across waking and sleep state, several investigations aimed to overcome the boundaries between different states of consciousness directly influencing sleep mentation by different kinds of sensory stimuli administered pre‐ or during sleep. Pre‐sleep stimulation methods have been used since the very beginning of dream research. The pioneering study by Dement and Wolpert (1958) showed the relation between the 24‐h fluid restriction in participants and their subsequent REM dream content. Sensory stimulation through pre‐sleep visual stimuli affected dream content by using stressful films (Goodenough et al., 1965) or visual inverting prisms (Corsi‐Cabrera et al., 1986).

Concerning sensory stimulation delivered during REM or NREM sleep stages, early studies described the incorporation of meaning verbal stimuli (Berger, 1963; Hoelscher et al., 1981). Also, somatosensory stimulation (e.g., water on the skin, thermal stimulation, pressure cuff, electrical pulses) (Baldridge et al., 1965; Dement & Wolpert, 1958; Koulack, 1969; Nielsen, 1993) or vestibular stimulation (Leslie & Ogilvie, 1996) were found to affect dream content. As expected, these types of stimulation increased vividness and bodily sensation in the dream contents.

Recent studies using olfactory stimulation during sleep showed the influence on the emotional content of dreams as a function of the hedonic characteristic of stimuli (Schredl et al., 2009) and the reactivation of the odour‐associated images (Schredl et al., 2014). The strong effect of olfactory stimulation on dream emotional aspects is interpreted in terms of direct connections to the limbic system (Smith & Shepherd, 2003).

In the last few years, a promising field of research explored the shared neural circuits between wake and sleep mentation by directly manipulating dream activity via transcranial electrical stimulation techniques. Some studies showed that interfering with cortical areas that are notably involved in a specific function during wakefulness influenced the dream content accordingly (Jakobson et al., 2012; Noreika et al., 2020).

Taken together, these results strengthen the hypothesis of shared mechanisms between the awake and sleeping brain from both psychological and neurobiological perspectives and through experimental manipulations. However, the intrinsic restraint due to the impossibility of directly investigating the dream content represents a common limitation of these studies.

### 1.4 What about direct access to dream experience?

The issue concerning dream access is definitively the most complex to address. Indeed, the real object of study in the abovementioned investigations (e.g., Chellappa et al., 2011; Marzano et al., 2011; Scarpelli et al., 2015, 2017; Scarpelli et al., 2020a; Scarpelli et al., 2019b; Siclari et al., 2017) is “dream recall” and not the dream experience *itself*. In other words, dreaming is not directly observable, and researchers are able to obtain information about the oneiric activity just requiring a dream report to the individual when he is awake. Also, we have already discussed that detecting the exact moment in which the dreams are produced during sleep is very difficult.

From a methodological point of view, three approaches to collect dreaming are well‐known: (a) retrospective, (b) prospective, and (c) provoked awakenings with subsequent dream reports. While the retrospective method allows researchers to collect dreaming through interviews or questionnaires in large samples quickly, the prospective protocol (i.e., dream diaries; longitudinal dream report collection) is less prone to memory biases (Robert & Zadra, 2008). These two strategies allow classifying people in high and low recallers, helping to investigate the neurobiological trait‐like features of dreamers (e.g., Eichenlaub et al., 2014b; Eichenlaub et al., 2014a; Ruby et al., 2021; van Wyk et al., 2019). However, the most accurate approach is represented by the provoked awakenings associated with the polysomnography (PSG) of one or more sleep nights in a laboratory. Generally, participants are awakened to explore the presence of a dream report and to compare the recall and non‐recall condition (Scarpelli et al., 2017; Scarpelli et al., 2020a; Siclari et al., 2017) or the report's qualitative features (Scarpelli et al., 2020b), correlating them with the specific EEG patterns preceding the awakening. It is worth noting that the narration of dream contents could be influenced by many biases after awakenings, such as the experimental setting (Schredl, 2008), the physiological background of waking‐life and by individual variables, such as personality, cognitive functions, censure/omissions and socio‐cultural features (Nir & Tononi, 2010), making dream reports not always completely reliable.

*How can we overcome this obstacle?* In this regard, recent studies have suggested that viable access to mental sleep activity is represented by dream‐enacting behaviours (DEBs; Baltzan et al., 2020). Any acting out of a dream during sleep characterised by motor, emotional or verbal components may be considered a direct observation of dream experience while the subject is asleep (Nielsen et al., 2009). In this view, the study of parasomnias or parasomnia‐like events, i.e., REM behaviour disorder (RBD), sleep walking, nightmares, and sleep talking, may provide new insights about dreaming. Interestingly, some investigations highlighted a strong level of congruence between the body movements, verbal or emotional expressions during sleep and the subsequent components of dream recall (Arkin et al., 1970; Leclair‐Visonneau et al., 2010; Oudiette et al., 2009; Rocha & Arnulf, 2020).

Assessing REMs in patients with RBD, Leclair‐Visonneau et al. (2010) found a concordance between limbs, head, and eye movements during the REM behaviour episode. The authors suggested that REMs may imitate the scanning of the dream scenario according to the so‐called “scanning hypothesis” (Arnulf, 2011; Leclair‐Visonneau et al., 2010). Moreover, Oudiette et al. (2009) revealed that during sleepwalking or sleep terror episodes, subjects show complex motor behaviours strictly related to their oneiric scenes. The same group has demonstrated that sleepwalkers are able to replay the recently trained behaviour during the parasomnia episode, supporting the idea that dream enactment may have a pivotal role in memory processing during sleep (Oudiette et al., 2011).

More recently, Rivera‐García et al. (2019) investigated the activation of facial muscles during REM sleep among healthy women. They considered facial expressions during sleep on a par with DEBs and an index of emotional dreams. Consistently, the previous literature shows that DEBs are more frequent during intense emotional dreams, such as nightmares (Nielsen et al., 2009). Indeed, the authors revealed that the activation of corrugator and zygomatic muscles are highly associated with dreams featured by negative affect (Rivera‐García et al. (2019)).

Also, sleep talking could be considered an additional non‐pathological parasomnia‐like event related to dreaming (Alfonsi et al., 2019; Mangiaruga et al., 2021). During sleep, the audible verbalisations may represent access to oneiric contents (Arkin et al., 1970; Alfonsi et al., 2019). In this regard, some studies showed different degrees of correspondence between sleep talking and dreaming (Arkin et al., 1970; Rechtschaffen et al., 1962). Arkin et al. (1970) reported different orders of concordance between sleep speech and later dream reports. Some authors investigated the presence of dialogical components within the dream reports proposing an overlapping between the neural mechanisms underlying linguistic production in dreams and those responsible for language during waking state (Shimizu & Inoue, 1986; Hong et al., 1996; Siclari et al., 2017). Specifically, Hong et al. (1996) found a reduction of the alpha activity focussed on Broca's and Wernicke's language regions, proportional to the amount of expressive and receptive language reported in dreams (Hong et al., 1996; Shimizu & Inoue, 1986). In addition, Noreika et al. (2015) demonstrated a decrement in the theta and alpha activity in a single‐case study associated with linguistic hypnagogic hallucination. Consistently, a recent study revealed that similar EEG patterns predict intelligible verbalisations during sleep (Mangiaruga et al., 2022).

Overall, both findings in subjects suffering from parasomnias and those related to “benign” phenomena (e.g., facial expressions, sleep talking), suggest that parasomnia‐like episodes may open a new frontier in dream research making the oneiric production more accessible.

#### 1.4.1 Nightmares

Nightmares are disturbing mental sleep activity characterised by negative emotions and often considered a clinical symptom causing significant distress. They are frequently associated with a high level of arousal and somatic manifestations that are capable to awake the dreamer from REM sleep. The repeated occurrence of this event is categorised as parasomnia, i.e., “nightmare disorder”, according to the Diagnostic and Statistical Manual of Mental Disorders, fifth edition (DSM‐5; American Psychiatric Association, 2013).

On the one hand, this disturbance is frequently related to post‐traumatic stress disorder (PTSD; Germain, 2013), but it could also be a reaction to stress conditions (Scarpelli et al., 2022). On the other hand, also idiopathic nightmares, i.e., without a known cause, should be considered. For instance, this kind of mental sleep activity is quite common in children tending to disappear during adulthood, and it is more frequent among females (Nielsen & Levin, 2007).

From a neurobiological perspective, a recent investigation shows that the activation of the autonomic nervous system may be linked to nightmares (Paul et al., 2019). Some studies revealed REM‐specific alterations in nightmare sufferers such as longer REM latency, increased skipping of early REM periods and cycle length, and more frequent REM periods (Nielsen et al., 2010). Furthermore, some EEG findings highlighted the presence of slow frontal and central theta activity during REM sleep in a group of nightmare recallers (Marquis et al., 2017). Further studies reported evidence for reduced slow‐wave sleep and greater intra‐sleep wakefulness (Simor et al., 2012), increased alpha power during REM sleep, and higher levels of EEG desynchronisation in NREM sleep of students with frequent nightmares (Simor et al., 2013). In other words, as already mentioned for dream recall, a higher autonomic and electrophysiological activation may provide the physiological background to the nightmare occurrence (Fisher et al., 1970; Nielsen & Zadra, 2005). This is consistent with the self‐reported experience of greater emotional and physical activations during the nightmare occurrence.

Fear is the predominant emotion included in nightmares (Zadra et al., 2006), suggesting that nightmares could be linked to fear‐dysfunction disturbances, i.e., phobias, generalised or social anxiety (Nielsen & Levin, 2007; Walker, 2010). In other words, nightmares could be related to the dysfunction in the hippocampal–amygdala prefrontal system that controls fear memory formation and extinction (Marquis et al., 2017; Nielsen & Levin, 2007). Nevertheless, the functional role of nightmares is still debated. Considering the early theories of dream function emphasising roles for REM sleep and dreaming in promoting adaptation to stress, nightmares could be interpreted as a failure of this process (Wright & Koulack, 1987).

Along this vein, some authors proposed that a certain degree of awareness of our dream contents and the possibility of altering them may be beneficial for nightmares sufferers (Kellner et al., 1992; Krakow et al., 2001; Neidhardt et al., 1992). In particular, compelling evidence highlighted that imagery rehearsal therapy (IRT) is very effective in reducing chronic nightmares within 6–12 weeks of therapy (Germain et al., 2004; Kellner et al., 1992; Krakow et al., 2001; Neidhardt et al., 1992). This technique consists of modifying the plot of the recurring nightmare during the wakefulness by an imaginal rehearsal of a new dream without disturbing items (Kellner et al., 1992). The nightmare sufferers learn to change the nightmares scenes by creating a less unpleasant ending and including mastery elements in the new dream scenario (Germain et al., 2004).

Interestingly, lucid dreaming induction could represent a useful intervention to reduce nightmares (Zadra & Pihl, 1997; Spoormaker & Van Den Bout, 2006; Rak et al., 2015). It has been hypothesised that lucid dreaming could be a sort of coping strategy to face unpleasant stimuli during a dream experience (Schiappa et al., 2018). Actually, lucid dream therapy is a cognitive technique that allows patients to learn to be aware of and modify their mental sleep activity during their nightmares through daily exercises (Spoormaker & Van Den Bout, 2006; Zadra & Pihl, 1997).

More recently, eye movement desensitisation and reprocessing (EMDR; Shapiro, 1989) has been employed for nightmares treatment in PTSD. Starting from the view that nightmares are the manifestations of adverse events registered in a dysfunctional form, this technique aimed to promote the recall of distressing images while activating one type of bilateral sensory input (e.g., hand tapping or side‐to‐side eye movement). The protocol allows subjects to identify and reprocess the targeted disturbing memories and experiences in order to formulate insight and adaptive behaviour.

In conclusion, it should be underlined that studies on PSG abnormalities and specific macro‐ and micro‐structural features correlated to nightmares are still missing. Further, efficacy studies on nightmare treatment (i.e., IRT, lucid dream therapy, EMDR) are scarce and fragmentary. Future research should be conducted to fill this gap and explore the effectiveness of the above‐mentioned interventions for nightmare disorders.

#### 1.4.2 What role for dreamwork in modern psychotherapy?

An interesting open issue concerns the possible usefulness of the oneiric experience as a tool in clinical practice, also in light of the neuroscientific knowledge on dreams.

Classically, Freud (1953) proposed two main functions of dreams: the expression of repressed infantile wishes and the protection of sleep. The antimoral nature of such wishes implies the need of a distortion through the dream censor to be acceptable, allowing their partial expression while protecting the continuity of sleep. Freud distinguished the manifest and the latent content of dream, the latter containing the true meaning of the dream. Free associations would represent the “royal road” to uncover the latent dream content, and the analyst provide his/her dream interpretation on the basis of the patient's dynamics.

The role of dream interpretation in modern psychoanalytic models has been significantly redefined compared to the initial Freudian conceptualisation (Pesant & Zadra, 2004). Crucially, several authors focussed their attention to the intrinsic validity of the manifest facets of dreams and their relationship with the diurnal experience. According to different approaches, the role of dream has been conceptualised in terms of reorganisation of the experience (Fosshage, 2002), adaptation to reality (Gazzillo et al., 2020), and co‐construction of the intersubjective reality (Jiménez, 2012).

Although several authors underline a “marginalisation” of dream in modern clinical psychological practice (Leonard & Dawson, 2018), it is worth noting that dreams have become an object of study also in clinical paradigms different from the psychoanalytical models (Pesant & Zadra, 2004; Velotti & Zavattini, 2019). Among the others, the evolution of the debate about dreaming in the cognitivist framework (Rosner et al., 2004) represents an interesting example of the redefinition of dreamwork in psychotherapy based on novel experimental data, theoretical models, and clinical observations. Beck (1971) proposed that dreams reflect the individual conception (and biases) about the self, the world, and the future, and may represent and indicator of changes in the emotional status. Nevertheless, the initial need to move away from the psychoanalytical framework and the pressure to adopt an empirically verifiable clinical model led to a common disuse of oneiric activity in cognitive‐behavioural psychotherapy. Dreams were mainly considered as psychologically meaningless epiphenomena of sleep, useless for the dreamer and in turn for the therapeutic process. More recently, the progress in the scientific understanding of dreams has led to the reintegration of dreams among the object of interest from different epistemological paradigms in the cognitivist framework. From a rationalist perspective, starting from the hypothesis that dreams are subjected to the same cognitive distortions that characterise the waking experience, it has been proposed that dreamwork can help to detect cognitive biases and maladaptive thought patterns (Barrett, 2002; Freeman & White, 2002; Hill, 1996, 2003) and promote cognitive reconstructing. On the other hand, the constructivist paradigm moved the focus on the narrative facets of dreams and the co‐construction of meaning between patient and therapist (Bara, 2012; Rezzonico & Bani, 2015; Rosner et al., 2004), with the aim to promote the emergence of relevant aspects of the personal meaning and increase the level of awareness of the patient.

The interest in the clinical use of dreams led to the development of different articulated models of dreamwork in psychotherapy, like the Description, Memory Sources, and Reformulation (DMR) model (Montangero, 2009) and the cognitive‐experiential model (Hill, 1996, 2003). Overall, Eudell‐Simmons and Hilsenroth (2005) identify four main functions of dreams in psychotherapy: (a) facilitate the therapeutic process, (b) increase patient insight and self‐awareness, (c) provide clinical information relevant for the therapist, and (d) provide a measure of therapeutic change.

Clearly, a further research effort is needed to provide support for the objective and efficacy of dreamwork in psychotherapy. Nevertheless, the ongoing debate on this topic has led to several models of the clinical valence of dreams that appear consistent with experimental findings on oneiric activity, mainly moving from standardised symbolic interpretations of dreams to approaches based on the relationship of dreaming with individual experience and cognitive/emotional/behavioural functioning.

## 2 CONCLUSIONS

From the discovery of REM sleep to the present day, empirical investigations have considerably increased our understanding of neural mechanisms underlying dream recall.

Although compelling evidence converges in providing support to the so‐called activation hypothesis and continuity hypothesis, considerable efforts are still needed to fully understand the neurobiological bases of oneiric processes.

Overall, we believe that (a) some results are still heterogeneous due to the application of different protocols, so a more consistent approach is needed; (b) the use of advanced techniques such as high‐density EEG or source localisation methods should be encouraged to better understand the relationship between specific oscillations and dream features; (c) further studies on experimental manipulation of dreaming should be carried out, also considering the implementation of brain stimulation techniques to promote dream recall or its specific characteristics; and (d) DEBs could be used as a model to observe dream contents overcoming the problem regarding the correspondence between specific time/stage of sleep and dream production, offering new insights about the neural correlate of dreaming.

Lastly, it is worth noting that recent pandemic studies have “elected” dream activity (and nightmares) as a reliable index of our emotional and psychological health (Fränkl et al., 2021; Scarpelli et al., 2022). Considering this, we underline that a translational view is needed to systematically explore the potential role of neurobiological and experiential facets of dreaming in a clinical context.

## AUTHOR CONTRIBUTIONS

All the authors contributed equally.

## CONFLICT OF INTEREST

All authors report no conflict of interest.

## Data Availability

N/A
